# A Wearable Multimodal Assistive Interface for Virtual Cursor Control in Stroke Survivors with Upper-Limb Impairment

**DOI:** 10.3390/s26175436

**Published:** 2026-08-28

**Authors:** Yuankai Liang, Liying Zhang, Ya Jiang, Junbiao Zhu, Yawen Zhao, Pengmin Qin, Di Chen, Junze Peng, Yuanqing Li, Xiquan Hu

**Affiliations:** 1 School of Automation Science and Engineering, South China University of Technology, Guangzhou 510641, China; 202210183128@mail.scut.edu.cn (Y.L.); aujiang.ya@mail.scut.edu.cn (Y.J.); aujubizhu@mail.scut.edu.cn (J.Z.); audidi2020@mail.scut.edu.cn (D.C.); 2 Research Center for Brain-Computer Interface, Guangdong Laboratory of Artificial Intelligence and Digital Economy (Guangzhou), Guangzhou 510335, China; qin.pengmin@m.scnu.edu.cn; 3 Department of Rehabilitation Medicine, The Third Affiliated Hospital, Sun Yat-Sen University, Guangzhou 510630, China; zhangly29@mail.sysu.edu.cn; 4 South China Brain-Computer Interface Technology Co., Ltd., Guangzhou 510320, China; 5 Department of Rehabilitation Medicine, Guangzhou Dongsheng Hospital, Guangzhou 510500, China; emir.321@hotmail.com; 6 Guangdong Key Laboratory of Mental Health and Cognitive Science, Key Laboratory of Brain, Cognition and Education Sciences, Ministry of Education, Center for Studies of Psychological Application, South China Normal University, Guangzhou 510631, China; 7 School of Computing and Data Science, The University of Hong Kong, Hong Kong 999077, China; u3656749@connect.hku.hk

**Keywords:** wearable assistive interface, multimodal interaction, electrooculography, electroencephalography, inertial measurement unit, virtual cursor, stroke rehabilitation, human–computer interaction, assistive technology

## Abstract

**Highlights:**

**What are the main findings?**

- A wearable multimodal assistive interface integrating electrooculography (EOG), electroencephalography (EEG), and inertial sensing was developed for virtual cursor control in stroke survivors with upper-limb impairments.
- The system supported reliable computer interaction across common daily tasks in a cohort of 30 stroke survivors, achieving an average operation accuracy of 87.53 ± 4.92% with a relatively moderate subjective workload.

**What are the implications of the main findings?**

- The findings demonstrate the feasibility of multimodal wearable sensing as an assistive approach for computer interaction in stroke survivors with upper-limb impairments.
- The user-specific calibration and multimodal control design offer a practical way to simplify system setup and support daily computer interaction.

**Abstract:**

Stroke survivors with upper-limb impairments often have difficulty using conventional computer interfaces, which limits their ability to perform daily computer-related activities independently. This study developed a wearable multimodal assistive interface that enables computer interaction through a virtual cursor. A lightweight headband equipped with electrooculography (EOG), electroencephalography (EEG), and an inertial measurement unit (IMU) was used to acquire multimodal signals for interaction control. EOG signals were processed to detect voluntary blinks to generate clicks, head movements were mapped to cursor movements through IMU-based control, and frontal EEG signals were used to estimate attention as an auxiliary mechanism for command verification. A rapid user-specific calibration procedure was introduced to adapt blink-detection thresholds to individual EOG characteristics without requiring extensive training. Thirty stroke patients with upper-limb impairments participated in experiments involving common computer tasks, including news reading, video playback, and character spelling. The system achieved an average operation accuracy of 87.53 ± 4.92%, an average operation time of 3.49 ± 0.49 s, and an information transfer rate of 62.04 ± 15.93 bits/min in the spelling task. The mean NASA-TLX score was 32.1 ± 5.4, indicating a moderate subjective workload during system use. These results demonstrate the feasibility of the proposed wearable multimodal assistive interface for supporting computer interaction in stroke survivors with upper-limb impairments.

## 1. Introduction

Stroke is a common disorder that is often associated with motor impairments, particularly upper-extremity dysfunctions such as hemiparesis and impaired motor control [1,2]. These impairments can limit physical function and make independent self-care activities difficult [3,4]. Maintaining independence in daily activities is important for these individuals, as it is closely related to their quality of life and psychological well-being [5,6]. Assistive technologies that allow patients to control external devices have been explored to support daily activities and improve independence [7,8]. Among these applications, restoring access to computers is important because computer interaction has become an essential part of modern life [9]. However, patients with upper-limb impairments often face difficulties when using conventional human–machine interfaces (HMIs), such as standard mice and keyboards, which can limit their ability to interact with computers effectively [10].

Eye trackers are widely used as alternative HMIs by generating control commands from users’ eye movements [11]. They have been applied to assist individuals with disabilities in accessing computers and browsing the internet independently, with promising results [12]. However, several limitations remain. Eye-tracking systems typically require users to maintain continuous visual control for extended periods, which may cause eye fatigue and affect interaction reliability during prolonged use [13]. In addition, stroke-related impairments may also affect eye movement control [14]. Environmental factors, such as lighting conditions, can further influence tracking accuracy, and patients who are bedridden for long periods may require additional eye care due to increased sensitivity to light exposure [15]. Therefore, eye trackers may not be an optimal HMI solution for all stroke patients with upper-limb impairments.

Among these approaches, wearable brain–computer interface (BCI)-based systems have been explored as another promising HMI approach, enabling users to control external devices or interfaces through neural signals. Previous studies have investigated the use of BCIs to improve computer accessibility for individuals with disabilities [16,17,18]. However, several challenges still limit their translation from experimental systems to practical applications. Many existing BCI studies primarily focus on improving control performance, while practical factors such as user comfort, ease of use, and clinical applicability remain important challenges [19]. This may result in discomfort during prolonged operation and reduce user acceptance. In addition, the interaction capability of current BCI systems is often restricted by their limited compatibility with common computing platforms and applications. For patients with motor impairments, an ideal HMI should support widely used devices, such as personal computers (PCs) and smartphones, while enabling diverse interaction functions, including clicking and text input. However, many BCI systems are designed as standalone platforms, making it difficult to integrate them into existing computer environments [20]. Furthermore, BCI performance may vary among users due to intersubject differences in electroencephalogram (EEG) patterns, which affects the stability and reliability of neural signal decoding [21]. Multimodal BCIs that combine multiple signal sources may help reduce the impact of such variability [22], but the resulting increase in system complexity remains a challenge for clinical deployment [23].

To address these limitations, this study developed a user-friendly virtual cursor system based on a lightweight wearable multimodal interface that integrates EOG, IMU, and frontal EEG signals to assist stroke patients with upper-limb impairments in computer interaction. This system was designed to support common computer operations, including cursor navigation, clicking, and text input, using a simple calibration procedure and commercially available hardware. It was evaluated through a series of daily computer tasks performed by 30 participants, and both operational performance and subjective workload were assessed to examine its usability in practical scenarios.

## 2. Materials and Methods

### 2.1. Participants

This study included 30 patients who had experienced stroke for 4.10 ± 3.38 months: 23 males and 7 females with an age range of 23–83 years (mean age: 50.63 ± 13.45 years). All participants had unilateral or bilateral upper-limb impairments, motor dysfunction, or reduced motor ability caused by stroke. The mean Fugl-Meyer assessment score for upper-limb motor function was 22.05 ± 5.90, with scores ranging from 14 to 31 out of a maximum of 34 [24]. The mean Brunnstrom recovery stages were 3.00 ± 1.26 for the upper extremity and 2.55 ± 1.54 for the hand [25]. These clinical assessments indicated varying degrees of residual upper-limb motor impairment among the participants.

**Figure 1:**
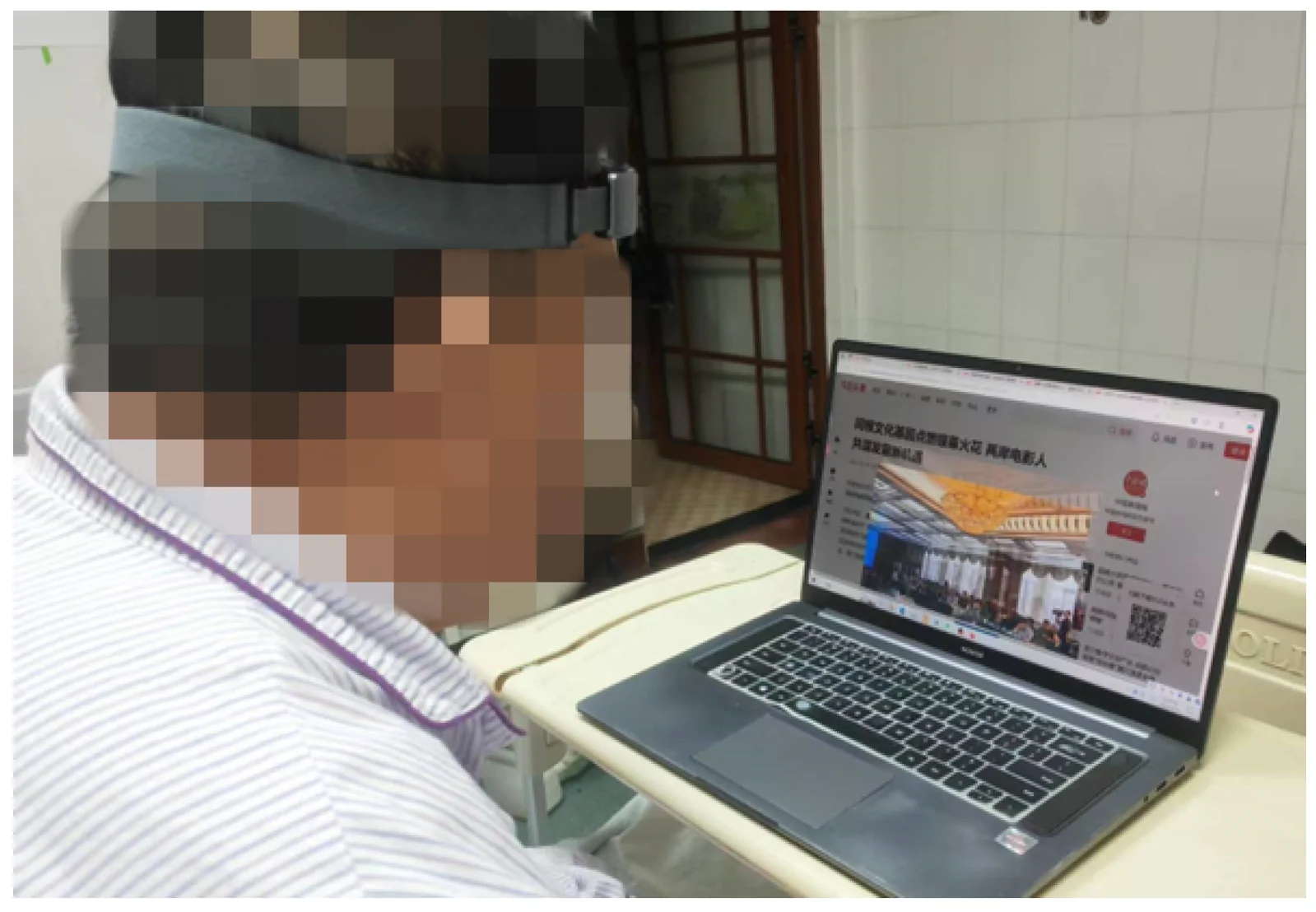
Participant using the system.

All participants had basic experience with computer use, but no prior experience with assistive computer interaction devices. They had sufficient head and eye movement abilities to operate the virtual cursor system and were able to understand and follow the experimental instructions. Written informed consent was obtained from all participants, and consent for the use of relevant clinical information was also obtained from their family members. To further protect participant privacy, images presented in this study were deidentified by blurring identifiable facial features. Figure 1 shows an example of a participant using the proposed virtual cursor system.

### 2.2. Equipment and Software

The wearable headband device (Isimple-TD03), developed by South China Brain-Computer Interface Technology Co., Ltd. (Guangzhou, China), contains one frontal EOG channel and two temporal channels (REF and DRL) equipped with replaceable hydrogel electrodes for real-time acquisition of electrooculography (EOG) and frontal EEG signals at a sampling rate of 250 Hz. The frontal electrode positioned near FP2 records EOG signals from the right frontal region, while the two lateral electrodes provide the reference (REF) and right-leg drive (DRL) signals, respectively. An inertial measurement unit (IMU) integrated with the headband, consisting of a three-axis accelerometer, gyroscope, and magnetometer, is used to measure the user’s head pose in real time [26]. The headband also includes an adjustable strap to maintain stable contact and improve wearing comfort. The total weight of the device is approximately 60 g.

After establishing a Bluetooth 4.2 connection, the accompanying software displays a virtual cursor on a PC or smartphone screen. The system supports multimodal interaction by combining EOG-based click commands, IMU-based cursor movement, and EEG-assisted command verification. In addition to basic cursor movement and clicking, the virtual cursor provides additional functions, including text input, long pressing, and scrolling through dedicated buttons. The cursor sensitivity can be adjusted according to individual differences in head-movement ability. The wearable device acquires EOG, IMU, and frontal EEG signals, which serve complementary roles during system interaction. Detailed signal processing and decision-making procedures are described in the following section.

### 2.3. Multimodal Signal Processing

The proposed multimodal system combines EOG, IMU, and frontal EEG signals to support multimodal interaction. EOG signals are processed to detect blink events and generate click commands, while IMU signals are used to translate head movements into cursor movements. Frontal EEG signals provide auxiliary information about the user’s cognitive state for command verification.

#### 2.3.1. EOG-Based Blink Detection

EOG signal segments were extracted using a sliding-window approach with a window duration of 1.2 s and a step size of 0.1 s. At the original sampling rate of 250 Hz, each segment contained 300 data points. To reduce computational cost while preserving the main waveform characteristics of blink events, the segments were downsampled to 125 Hz, resulting in 150 data points per segment. A binomial smoothing filter with the kernel [1, 4, 6, 4, 1]/16 was then applied to attenuate high-frequency noise in the raw signals [27].

Baseline drift is a common low-frequency artifact in EOG recordings [28]. To reduce the influence of this artifact, a sliding-window local mean method was applied. The corresponding mathematical formulation and processing steps are described as follows:

(1) $$B\left[i\right]=\frac{1}{N_{i}}\Sigma_{k=Start\left[i\right]}^{End\left[i\right]}x\left[k\right]$$

where $x\left[k\right]$ is the k-th sampling point of the filtered signal; Start[i]=max(1,i−floor(W/2)) and End[i]=min(L,i+floor(W/2)) are the start and end index of the sliding window for the i-th sampling point; W=250 is the sliding-window size selected in this algorithm (corresponding to a two-second-long sample); L is the total length of the filtered signal; $N_{i}=End[i]-Start[i]\;+\;1$ is the actual number of sampling points within the sliding window at position i (ensuring that the baseline estimation adapts to the signal boundaries); and B[i] is the estimated local baseline value for the i-th sampling point.

Therefore, the baseline-corrected signal $\hat{x}$ can be expressed as:

(2) $$\hat{x}\left[i\right]=x\left[i\right]-B\left[i\right]$$

**Figure 2:**
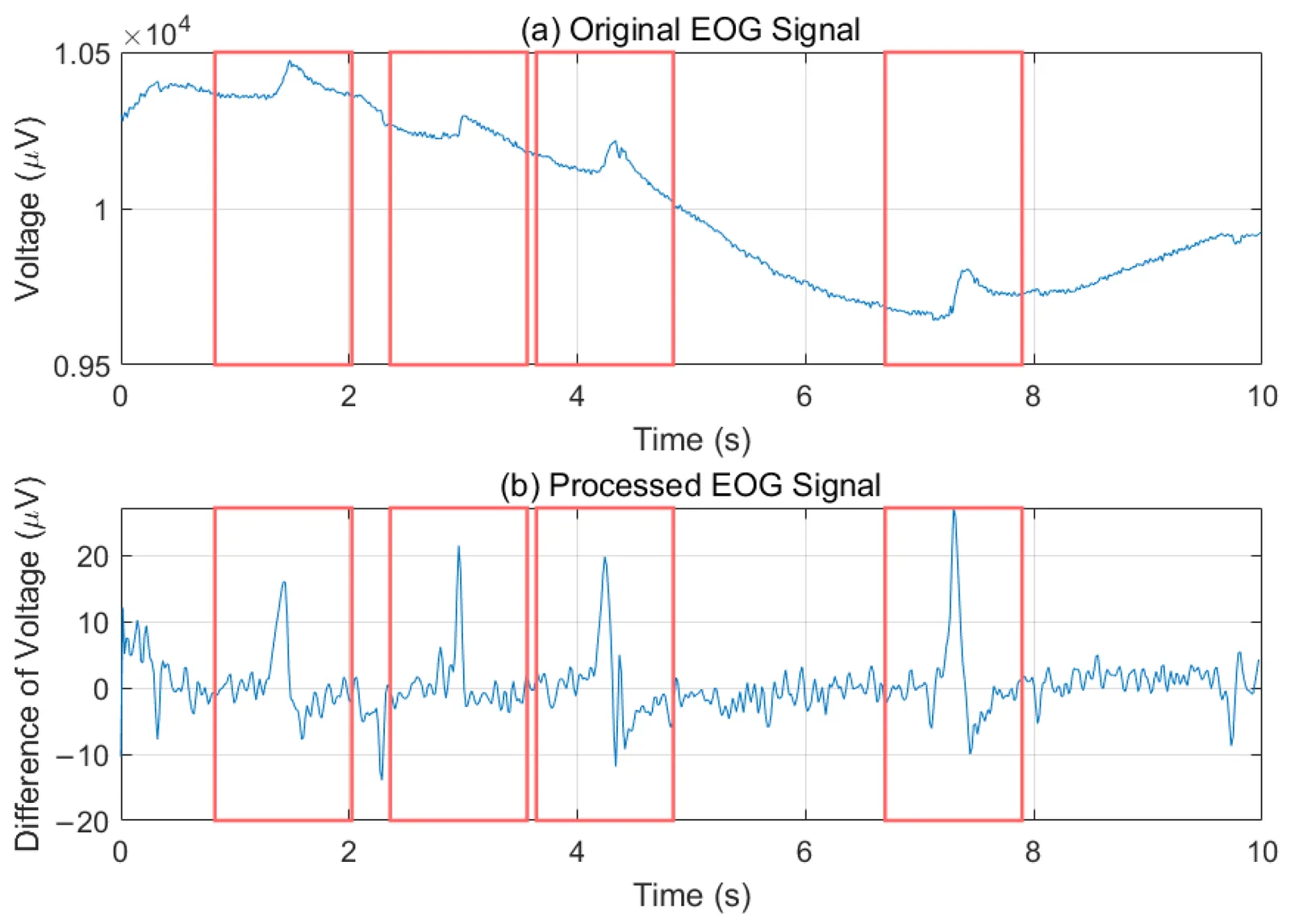
(**a**) The original EOG signal. (**b**) The target EOG signal after processing. The blink signals are highlighted with red boxes.

After baseline correction, a first-order difference was applied to the baseline-corrected EOG signal $\hat{x}$ to emphasize the rapid waveform changes associated with blink events. For a baseline-corrected signal segment of length 150, the resulting differenced signal contains 149 sampling points. The resulting signal ${\hat{x}}^{\prime }\;$was used for subsequent feature extraction and blink detection. An example of the processed signal is shown in Figure 2.

Following signal preprocessing, including baseline correction and first-order differencing, time-domain features were extracted from EOG segments to characterize blink-related waveform patterns. Previous studies on EOG-based blink detection have commonly used amplitude, temporal, slope, energy, and statistical features to distinguish blink-related waveforms from non-blink events [29,30,31]. Accordingly, ten candidate features describing waveform amplitude, temporal characteristics, statistical properties, and energy were considered in this study. These features were evaluated using EOG recordings from 30 participants to identify suitable feature combinations for blink detection. For a signal segment represented as $x=\left(x_{1},\ldots ,x_{n}\right)$, where n=149, these features are defined in Table 1.

**Figure 3:**
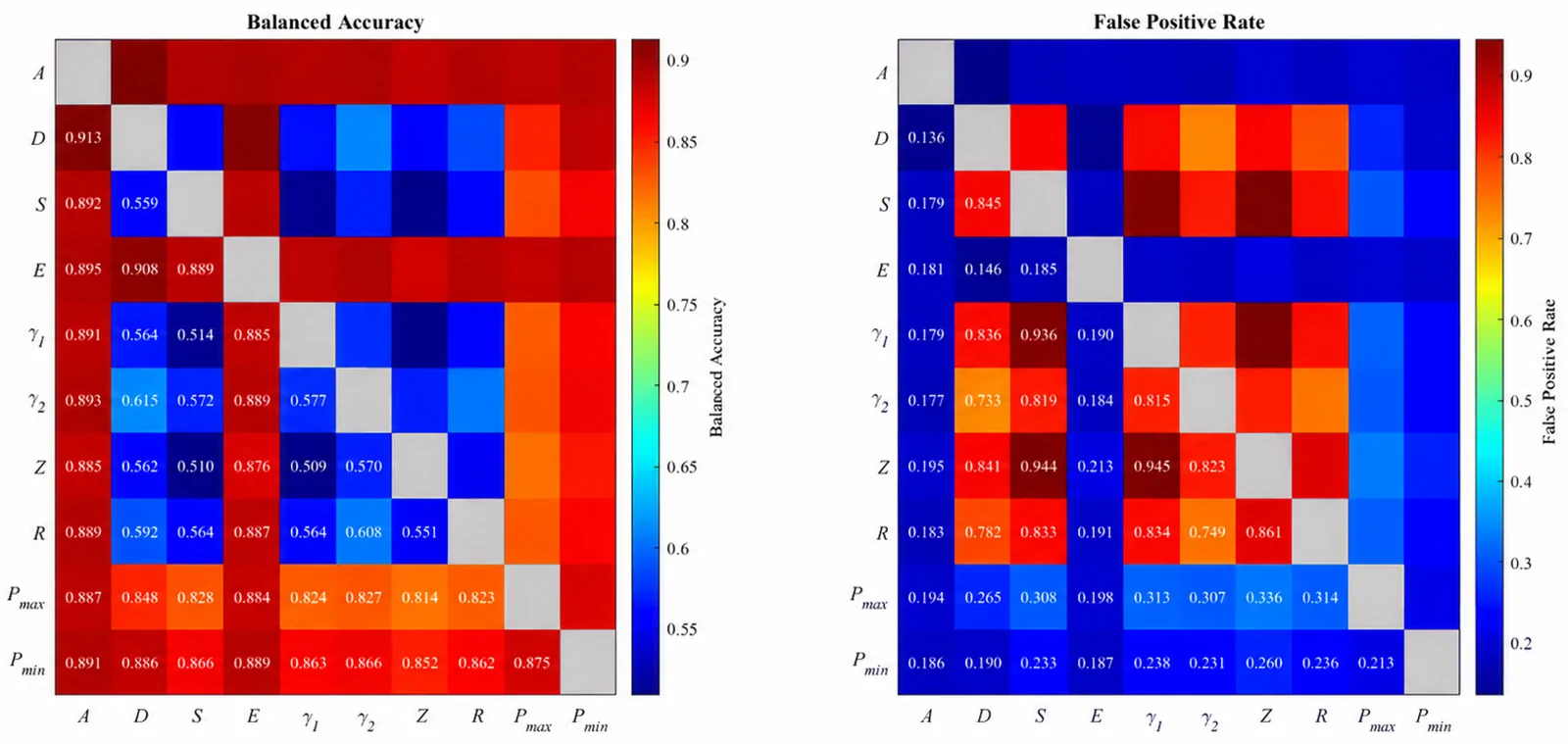
Matrices of average balanced accuracy and false-positive rate of feature combination.

For each feature combination, a fivefold cross-validation procedure was performed. In each fold, feature boundaries were estimated only from the training blink samples using the 1st and 99th percentiles to reduce the influence of outliers, and the detection performance was evaluated on the remaining samples. The combination of peak-to-trough amplitude (A) and peak-to-trough distance (D) achieved a favorable balance between balanced accuracy and false-positive rate and was selected for online implementation. The average balanced accuracy and false-positive rate of each feature combination across fivefold cross-validation are shown in Figure 3.

The percentile-based evaluation was used only for offline feature selection and performance comparison where multiple samples from different participants were available. However, during practical operation, only a short user-specific calibration procedure is available, so all calibration trials were retained to determine individual detection boundaries.

Before online operation, each user completed a short calibration procedure. The user was instructed to perform 10 blinks while the system recorded the corresponding EOG waveforms. For the i-th blink, the feature values $A_{i}$ and $D_{i}\;$were calculated. The upper and lower boundaries of each feature were then determined from the maximum and minimum values, denoted $A_{max}$, $A_{min}$, $D_{max}$, and $D_{min}$, observed across the calibration trials.

(3) $$\left\{\begin{array}{c} A_{max}=\max\left(A_{1},\ldots ,A_{10}\right),\;A_{min}=\min\left(A_{1},\ldots ,A_{10}\right)\;\\ D_{max}=\max\left(D_{1},\ldots ,D_{10}\right),\;D_{min}=\min\left(D_{1},\ldots ,D_{10}\right) \end{array}\right.$$

During online operation, the result (r) of a blink event is detected only when both the amplitude A and distance D extracted from the real-time EOG segment fall within their corresponding calibration ranges:

(4) $$r=\;\left\{\begin{array}{c} 1,\;A_{min}\leq A\leq \;A_{max}\;and\;D_{min}\leq D\leq \;D_{max}\;\\ 0,otherwise \end{array}\right.$$

In addition, alternative statistical thresholding strategies were evaluated during offline evaluation. For example, thresholds based on the mean ± k·standard deviation of blink features were tested. However, these methods yielded lower balanced accuracy (<0.7) compared with the selected range-based strategy. The range-based calibration approach was therefore retained because it provided better separation between blink and non-blink events while requiring only a small number of calibration trials.

#### 2.3.2. IMU-Based Cursor Movement Control

Changes in head orientation are used to generate cursor movement commands. An IMU mounted on the user’s head continuously measures the head’s yaw and pitch angles. The IMU signals are first filtered using a low-pass filter to reduce high-frequency noise. Changes in the filtered yaw and pitch angles are then converted into horizontal and vertical cursor displacements, respectively.

Assuming that the head rotations during cursor operation are relatively small, the incremental changes in yaw (Δψ) and pitch (Δθ) are linearly mapped to cursor displacements on the screen. The horizontal cursor movement Δx is proportional to the yaw angle change Δψ, while the vertical cursor movement Δy is proportional to the pitch angle change Δθ. The corresponding cursor displacement is calculated as follows:

(5) $$\left\{\begin{array}{c} \Delta x=S_{x}\Delta \psi \;\\ \Delta y=S_{y}\Delta \theta \end{array}\right.$$

where $S_{x}$ and $S_{y}$ denote the cursor sensitivity coefficients, which convert angular changes into pixel displacement on the screen. The cursor position is updated cumulatively based on its previous cursor position:

(6) $$\left\{\begin{array}{c} x_{t+1}=x_{t}+\Delta x\;\\ y_{t+1}=y_{t}+\Delta y \end{array}\right.$$

**Figure 4:**
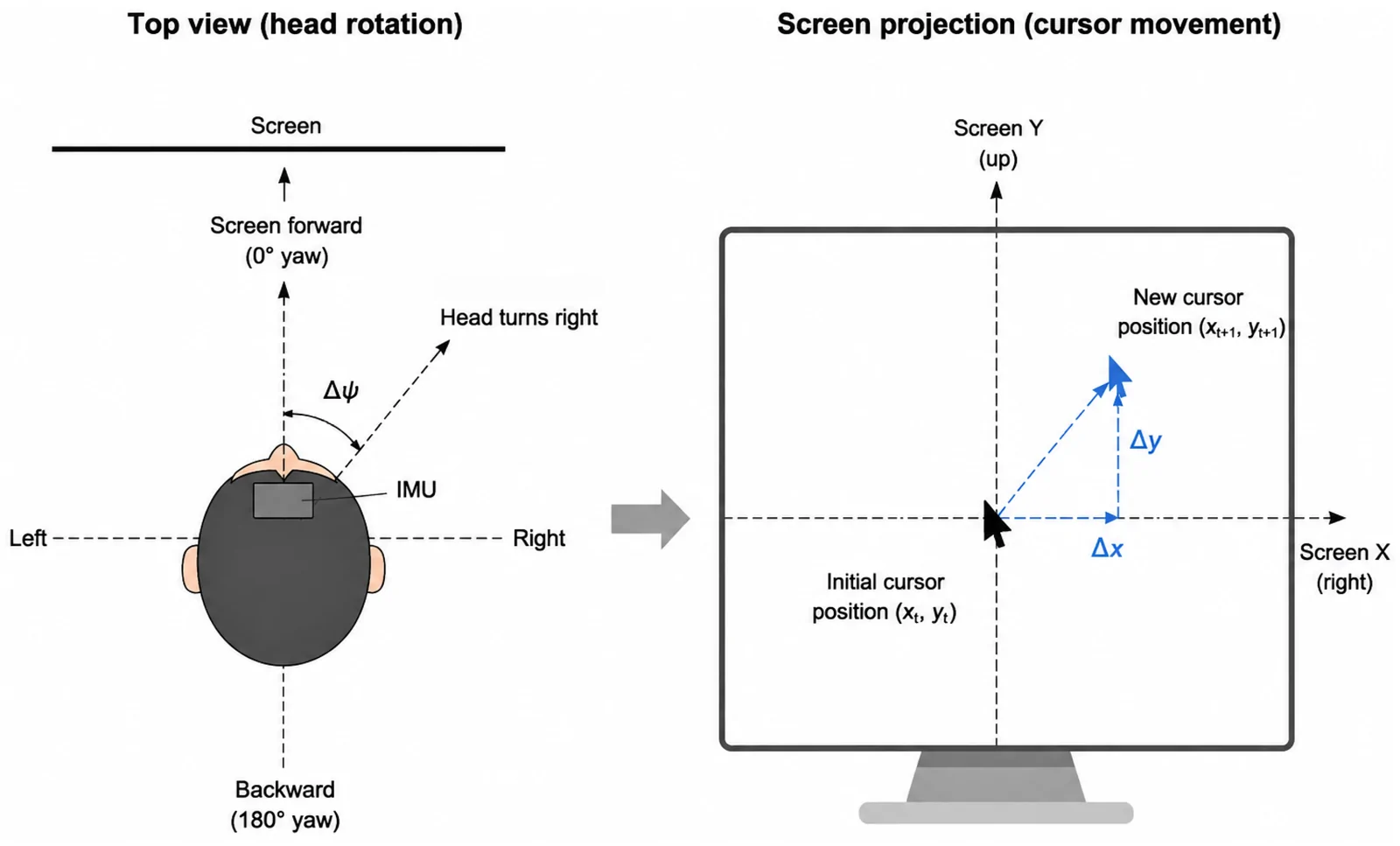
Schematic illustration of IMU-based cursor movement control. Blue dashed arrow on Screen projection indicate the cursor movement vector, with Δx and Δy representing the movement components along the X and Y axes.

Before each experiment, participants were allowed to adjust the cursor sensitivity according to their comfort and head-movement ability. A schematic diagram of IMU-based cursor control is shown in Figure 4.

#### 2.3.3. EEG-Based Attention Monitoring

In addition to EOG-based click detection and IMU-based cursor movement control, frontal EEG signals were used as an auxiliary measure of cognitive state for command verification [32]. The EEG signal was not used to directly generate interaction commands. Instead, an EEG-derived attention-related index was calculated to provide additional information for determining whether a detected click command occurred during an attentive state.

The frontal EEG signals were analyzed in the frequency domain using short-time Fourier transform (STFT) [33]. A 1 s Hanning window without overlap was applied to reduce spectral leakage. The average power spectral density (PSD) values within the theta (4–8 Hz) and beta (13–30 Hz) bands were calculated and denoted $E_{\theta }\left(t\right)\;$and $E_{\beta }(t)$, respectively [34]. Then, an attention index was calculated using the logarithmic ratio between theta and beta powers [35]:

(7) $$a(t)=log_{2}(\frac{E_{\theta }(t)}{E_{\beta }(t)})$$

where a larger a(t) value indicates a higher theta-to-beta power ratio and therefore corresponds to a lower attention state. To account for temporal variations in EEG characteristics arising from individual differences and changes in cognitive state, a dynamic threshold strategy was used. At the beginning of system operation, the EEG-based verification mechanism was initialized, and detected click commands were temporarily accepted without EEG-based rejection. The attention index was updated using a 5 s sliding window with a 1 s step, with each window producing one attention-index sample for continuous monitoring [36]. This 5 s window was used to calculate individual attention-index samples, whereas the subsequent threshold update was based on the 30 most recent attention-index samples, corresponding to approximately 30 s of recent history. After sufficient attention-index samples were collected, the threshold was updated according to the recent attention index:

(8) $$a_{reject}(t)=0.7\times \frac{\Sigma_{i=t-29}^{t}a(i)}{30}$$

During subsequent operation, a detected blink command was accepted only when $a\left(t\right)<\;a_{reject\;}(t)$. Otherwise, the command was considered to occur under a relatively low-attention state and was rejected as a potential unintended activation.

### 2.4. Experimental Procedures and Performance Evaluation

During the experimental period, participants maintained their usual daily routines to keep their rehabilitation conditions consistent. Each experiment consisted of a 10 min training session followed by a 20 min test session. During training, participants became familiar with the operation of the virtual cursor system. During the test session, they interacted with web pages or software applications through a set of computer tasks selected to reflect common daily computer use, including news reading, video playback, and character spelling.

**Figure 5:**
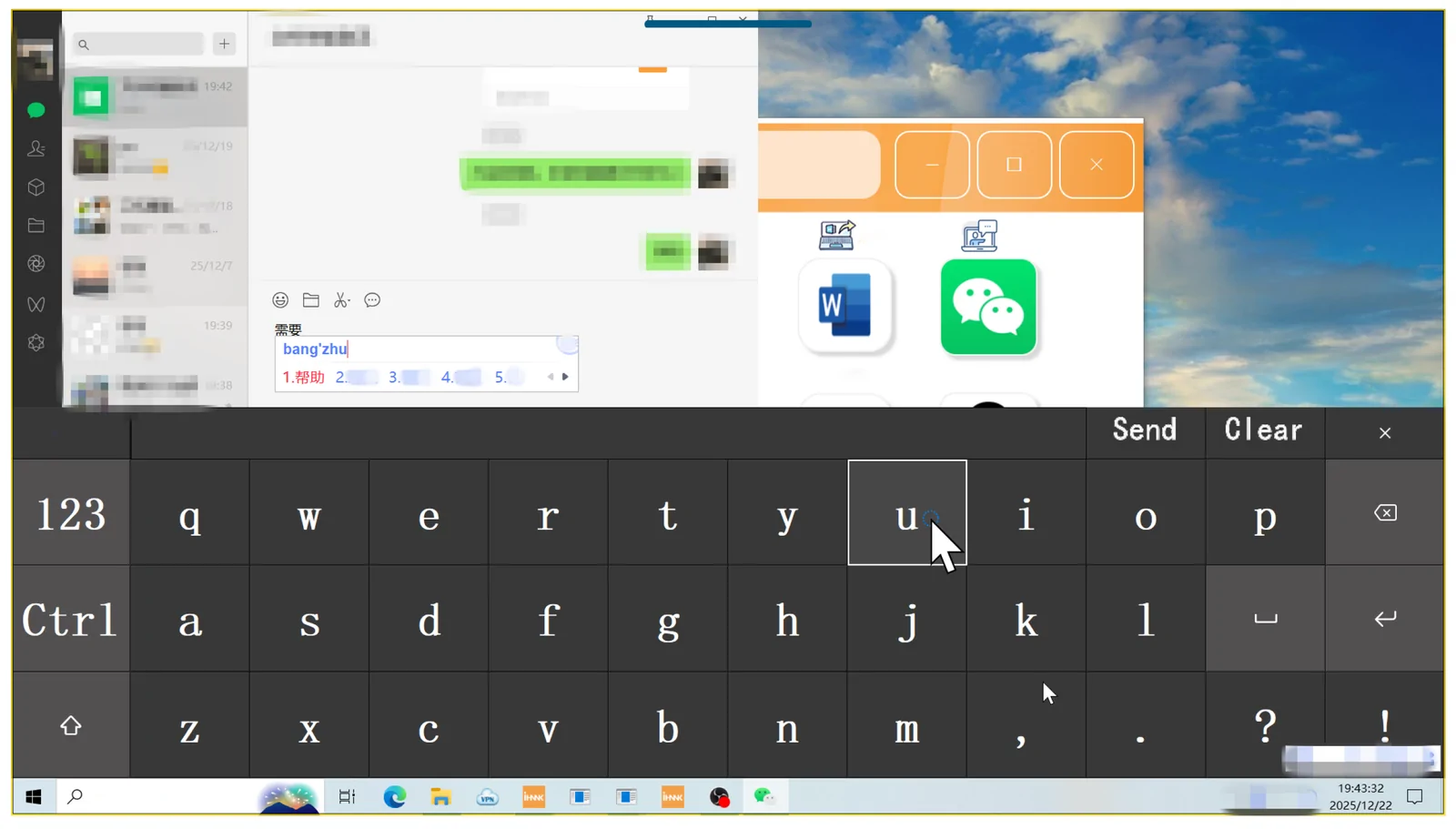
Example interface of the character spelling task using the virtual cursor system. In this scenario, the participant has already entered the word “需要” (“need”) and is about to select “帮助” (“help”) from the word options to enter it.

News reading and video playback represent typical internet activities, and were performed on general-purpose online platforms to maintain a realistic usage scenario. Character spelling was included because text input is an essential communication method and is also commonly used for evaluating interactive brain–computer interface systems. This task was performed using a virtual keyboard containing 29 buttons, through which participants entered Chinese phrases using Chinese pinyin. Participants were allowed to choose the phrases they wished to enter, with at least 10 English letters required during the character spelling task. For example, the phrase “我需要帮助” (“I need help”) was entered using the Pinyin sequence “*wo xu yao bang zhu*,” which contains 14 letters. Participants could press the space bar to select the first candidate provided by the virtual keyboard or click the desired candidate from the displayed options. For participants unfamiliar with Chinese pinyin, the researcher provided verbal instructions on which letters to select. An example interface of the character spelling task is shown in Figure 5.

System performance was evaluated at the operation level across the three tasks. A selection of the intended button was counted as a correct operation, whereas an unintended selection was counted as an erroneous operation. During character spelling, an erroneous selection was corrected by selecting the delete button before continuing the intended input. These operation-level results were used to calculate the operation accuracy and average operation time. In addition, the information transfer rate (ITR) was calculated for the character spelling task to characterize information transmission efficiency. The definitions of these performance metrics are provided in Table 2.

Subjective workload was evaluated separately to characterize participants’ perceived burden, satisfaction, and acceptance during system use. The NASA-TLX scale [37] was applied to assess workload across six dimensions: mental demand, physical demand, temporal demand, performance, effort, and frustration. In this study, the raw NASA-TLX score was calculated as the average rating across the six dimensions without applying weighting procedures.

**Table 2:** Performance metrics for system evaluation.

Experimental Metrics	Description
Operation accuracy	The proportion of correct operations among all operations in a task, defined as:
	$a=\frac{N_{correct}}{N_{correct}+N_{error}}$
	where $N_{correct}$ and $N_{error}$ denote the numbers of correct and erroneous operations.
Average operation time	The average time T required to complete a single operation, measured from the initiation of cursor movement to the completion of the corresponding action.
Information transfer rate (ITR)	The information transmission rate during the character spelling task [38], defined as:
	$ITR=\frac{1}{T}(\log_{2}N+alog_{2}a+\left(1-a\right)\log_{2}\frac{1-a}{N-1})$
	where a and T denote the operation accuracy and average operation time in the spelling task and N = 29 is the number of keys on virtual keyboard, including 26 letter buttons, space button, delete button, and send button.

## 3. Results

All 30 patients successfully completed the experimental tasks using the proposed virtual cursor system. The performance metrics were calculated based on their recorded operation sequences. During the test, patients completed an average of 47.07 ± 19.49 operations, with the number of operations ranging from 25 to 102. The average accuracy across all participants was 87.53 ± 4.92%, with nine patients achieving an accuracy above 90%. The maximum accuracy reached 97.14%. The average completion time per operation was 3.49 ± 0.49 s, and only five patients required more than 4 s per operation. The task-specific performance results are summarized in Table 3.

**Figure 6:**
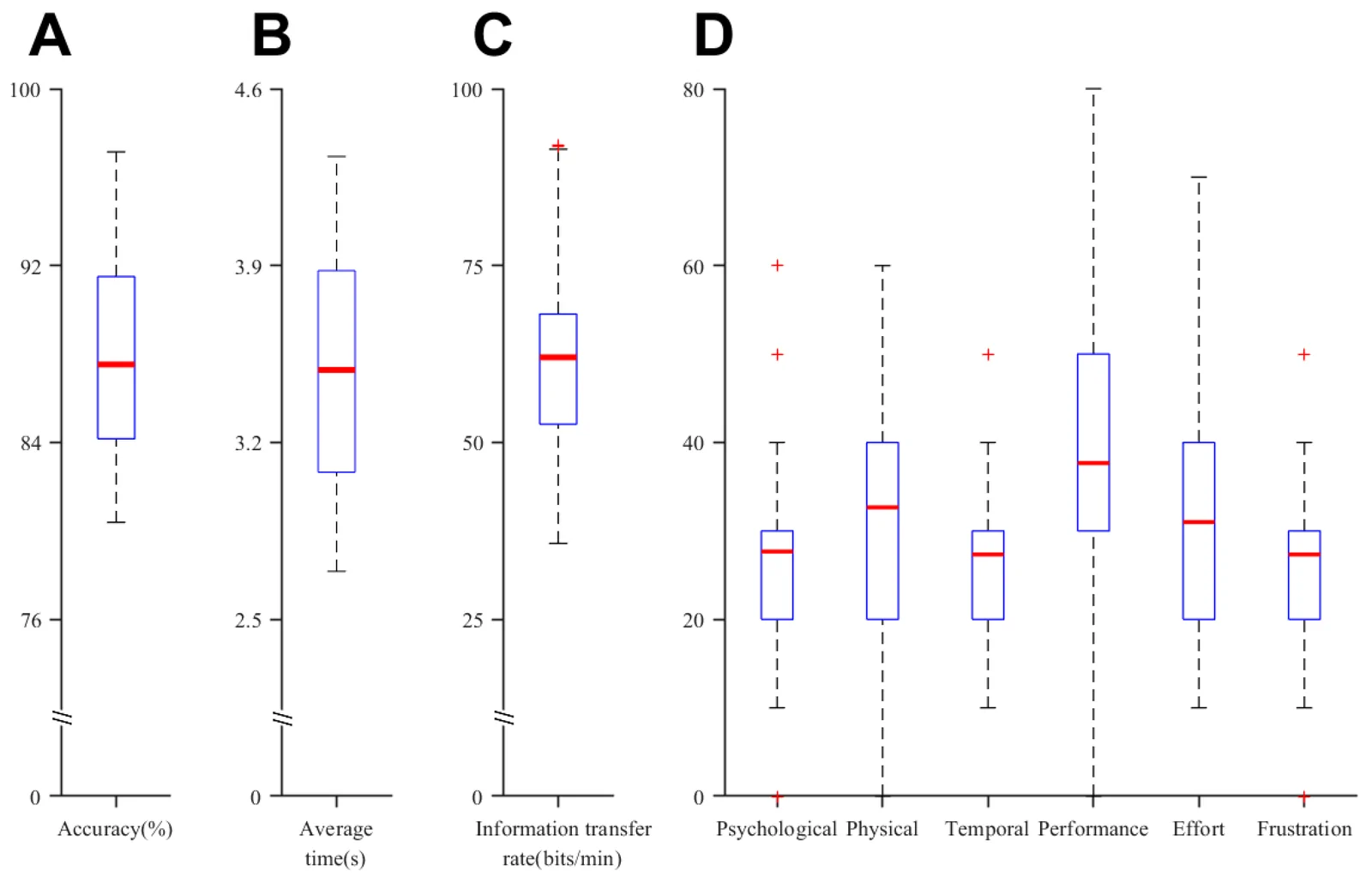
Evaluations of system performance and subjective workload. (**A**) Accuracy, (**B**) average time, (**C**) information transfer rate, (**D**) subjective workload.

For the character spelling task, the average accuracy and completion time were 85.32±11.87% and 3.62±0.82 s, respectively. The corresponding information transfer rate (ITR) was 62.04±15.93 bits/min on average. Four patients achieved an ITR above 90 bits/min, with the highest value reaching 92.08 bits/min. A detailed distribution of performance metrics is presented in Figure 6.

In Figure 6, the blue box plots show the distribution of results across participants. The red horizontal lines inside the boxes indicate the mean values. The boxes represent the range between the first and third quartiles, while the dashed lines extending from the boxes indicate values outside this range. Red crosses denote outliers.

Subjective workload was evaluated using the NASA-TLX questionnaire. The mean scores for the six subscales were mental demand (27.7 ± 12.8), physical demand (32.7 ± 14.8), temporal demand (27.3 ± 10.2), performance (37.7 ± 17.0), effort (31.0 ± 15.2), and frustration (27.3 ± 13.9). The overall mean workload score was 32.1 ± 5.4. Except for one participant who reported a performance score of 80 and another who reported an effort score of 70, all other participants reported subjective workload scores below 60.

Spearman rank correlation analysis was performed to investigate the associations between patient characteristics and system performance [39]. The correlation results are summarized in Table 4. The Fugl-Meyer score showed moderate positive correlations with operation accuracy (ρ=0.448, p=0.037) and ITR (ρ=0.439, p=0.041), whereas its correlation with average operation time was not statistically significant (ρ=0.220, p=0.326). No significant associations were found between disease duration and operation accuracy, average operation time, or ITR (all p>0.05). Similarly, neither the Brunnstrom recovery stage of the upper extremity nor that of the hand was significantly associated with any of the evaluated performance measures. Age was not significantly associated with any performance measure, although a moderate positive association with average operation time was observed (ρ=0.404, p=0.062).

## 4. Discussion

In this study, we developed a wearable multimodal virtual cursor system that integrates EOG, IMU, and frontal EEG signals to facilitate computer interaction for stroke patients with upper-limb impairments. Rather than relying on conventional hand-operated input devices, the proposed system allows users to perform basic computer operations through head movements and eyeblink-based clicking, with EEG-based attention monitoring providing an additional command verification mechanism. All 30 stroke patients successfully completed common online tasks, including news reading, video playback, and character spelling. The system achieved an average operation accuracy of 87.53 ± 4.92% and an average operation time of 3.49 ± 0.49 s, indicating that it can provide effective cursor control and computer interaction for stroke patients with preserved head and eye movement abilities.

The performance of the proposed system was comparable to that reported in recent studies of multimodal BCIs for individuals with motor impairments [40]. This result supports the potential of wearable hybrid BCI systems for daily computer interaction. In our previous study, patients with amyotrophic lateral sclerosis (ALS) achieved an average accuracy of 83.9% with an average response time of 5.12 s using a wearable BCI cursor system for similar computer tasks [26]. Compared with these results, the present system achieved similar accuracy with a shorter response time. This discrepancy may be related to differences in residual motor function and user characteristics between the two populations.

The associations between patient clinical characteristics and system performance were also examined. Higher Fugl-Meyer scores were moderately associated with higher operation accuracy and ITR, suggesting that participants with better residual motor function tended to achieve more accurate and efficient interaction. However, Fugl-Meyer score was not significantly associated with average operation time. Disease duration was not significantly associated with any of the evaluated performance measures, suggesting that the time elapsed since stroke alone may not be a major determinant of system performance in the studied cohort. Neither upper-extremity nor hand Brunnstrom recovery stage showed a significant association with system performance. Although age showed a moderate positive association with operation time, this relationship did not reach statistical significance. These findings suggest that residual motor function may affect some aspects of interaction performance, while the proposed system remained usable across a range of motor impairment levels represented in the study. Given the relatively small sample and exploratory nature of this analysis, these associations should be interpreted cautiously.

To achieve reliable blink detection while accommodating individual physiological differences, a brief calibration procedure was performed before system use. This procedure allowed rapid system setup without requiring extensive user training. During calibration, each participant performed 10 blinks, from which two time-domain features, peak-to-trough amplitude and peak-to-trough distance, were extracted. The minimum and maximum values of these features were then used to define the detection thresholds for each user. This threshold-based approach is straightforward to implement and easy to interpret, making the calibration procedure practical. Its effectiveness is supported by the experimental results, which show that the proposed system achieved stable performance with satisfactory detection accuracy across the patient cohort. Although min–max calibration may be sensitive to individual outliers, it was adopted because it provides a simple and user-specific calibration procedure requiring only a small number of blink trials. The robustness of the selected feature combination was additionally evaluated using EOG recordings from multiple participants, which showed an acceptable balance between blink acceptance and false-activation rejection compared with other candidate feature combinations.

To further understand the system performance, we analyzed the sources of operation errors. The observed errors were categorized into three types: false positives (unintended clicks), false negatives (missed clicks), and navigation errors (clicking at an incorrect location). False positives accounted for approximately 73.7% of all errors and were primarily caused by involuntary eye blinks or unintended head movements. This result suggests that improving the discrimination between intentional and unintentional blinks, for example, by further refining the attention-based filtering strategy or temporarily suppressing click detection during cursor movement, may further reduce false activations. False negatives accounted for only 6.82% of the recorded errors. They were typically observed when participants attempted to click immediately after moving the cursor without a brief pause, resulting in distorted EOG waveforms that were more difficult to detect reliably. Navigation errors occurred mainly when participants interacted with small interface elements, such as the “close” button of a web page. These errors were particularly common during the character spelling task because the virtual keyboard contained densely arranged keys, requiring more precise cursor positioning.

Although the dense key layout contributed to the lower ITR observed in the spelling task, it does not fully account for the substantial variation among participants. Several participants achieved an ITR above 90 bits/min, whereas others achieved less than 50 bits/min. Notably, all participants with an ITR below 50 bits/min had Fugl-Meyer scores below 20, while all participants with an ITR above 90 bits/min had scores above 26. Together with the moderate positive correlation between Fugl-Meyer scores and ITR, these findings suggest that residual motor function may affect performance in character spelling tasks. One possible explanation is that patients with more severe motor impairment may have had fewer opportunities to use computer-based text-entry functions after stroke, resulting in less familiarity with or practice using pinyin-based input. Individual factors, such as computer experience and familiarity with Chinese pinyin input, may also have contributed to the observed variation.

Clinical usability is an important consideration in the design of assistive systems and depends on both hardware and software [41]. The proposed hardware adopts a lightweight, wearable design that reduces physical burden and improves comfort during prolonged use [42]. In addition, key components, including the electrode modules and control buttons, are built with industrial-grade components, making the device easier to maintain and its functions easier to understand. These design choices reduce the learning effort required for system operation, allowing healthcare professionals and first-time users to guide patients with only minimal training.

The NASA-TLX results further characterized the subjective workload associated with system operation. In this study, raw NASA-TLX scores were used to assess perceived workload during interaction. Nearly all participants reported overall workload scores below 60, with an average score of 32.1, indicating that the proposed system imposed a moderate workload during use. NASA-TLX has been widely used to evaluate subjective workload in assistive and human–computer interaction tasks [43]. Reported workload levels can vary substantially with task complexity, control modality, and user population. For comparison, a previous study involving participants with motor impairments reported a raw NASA-TLX score of 39.8 ± 8.1 for a 1D horizontal cursor-control task [44]. The lower score observed in the present study suggests a lower perceived workload, although this comparison should be interpreted cautiously because of differences in task design and participant characteristics.

The software was designed to provide broad compatibility by implementing the interface as a virtual cursor that can operate across different operating systems. As a result, users only need to learn a single interaction method while continuing to use familiar software applications, reducing the learning burden and improving the practicality of the system. In addition, the personalized calibration procedure allows users to begin operating the system shortly after wearing the device. The calibration process requires approximately 30 s and establishes user-specific detection thresholds without additional training or complicated parameter adjustment, further improving usability and facilitating deployment in clinical practice.

The proposed system adopts a multimodal design that combines EOG, head movement, and EEG-based attention estimation to improve interaction reliability while maintaining a simple hardware architecture. Each modality has a distinct role, enabling cursor movement, click detection, and command verification to work together. One limitation of this approach is that attention estimation may become less reliable in the presence of large EOG fluctuations. Moreover, facial muscle activity, such as jaw clenching, or fatigue after prolonged use may introduce interference, increasing the likelihood of unintended clicks. Nevertheless, considering its low cost and consumer-grade hardware, the proposed system achieved satisfactory performance in practical computer interaction tasks [45].

Several limitations remain. Although the system was evaluated in 30 stroke patients, most participants had sufficient residual head and eye movements for interaction. During the evaluation, some patients with more severe motor impairment also attempted to use the system, but had difficulty operating it reliably because of involuntary head movements or limited head-movement range. Therefore, its applicability to patients with severe motor impairment requires further investigation. Unintended clicks occasionally occurred during operation, particularly due to spontaneous blinks or rapid consecutive clicks when interacting with small interface elements. These events could lead to unstable signal recognition and affect the overall user experience. The attention-based verification mechanism was designed to reduce unintended activations by incorporating auxiliary cognitive-state information. However, no ablation study was conducted to directly compare system performance with and without EEG-based attention verification, so its specific contribution to reducing unintended activations could not be quantified. In addition, the attention-based mechanism requires users to maintain a certain level of attention, and prolonged use may increase cognitive and physical fatigue. The formal evaluation was limited to approximately 30 min, including training and testing. Some participants subsequently reported increased fatigue during extended use in inpatient rehabilitation, but these observations were not collected using a standardized fatigue assessment scale. A systematic long-term usability evaluation is therefore needed in future studies. Despite these limitations, the results indicate that the proposed virtual cursor system provides a practical human–computer interaction solution for stroke patients with upper-limb impairments. Future studies will include patients with more severe motor deficits, directly compare system performance with and without EEG-based verification, and evaluate the system across different levels of impairment and longer periods of use.

## 5. Conclusions

Efficient human–computer interaction is important for improving the independence of stroke patients with upper-limb impairments. To address this need, we developed a lightweight wearable multimodal assistive interface that integrates EOG, EEG, and head-motion sensing for virtual cursor control. Evaluation in 30 stroke patients demonstrated that the system supported common computer tasks with an average operation accuracy of 87.53%, an average operation time of 3.49 s, and a mean raw NASA-TLX score of 32.1, indicating reliable interaction performance with a relatively moderate subjective workload. These findings demonstrate the feasibility of the proposed system as a practical assistive interface for restoring computer access in individuals with upper-limb impairments. Although unintended clicks, prolonged-use fatigue, and applicability to patients with more severe motor impairments remain challenges, the results support its potential for practical clinical use. Future work will focus on evaluating the system in patients with more severe motor deficits, further assessing the contribution of EEG-based verification, and extending the interface to additional assistive devices, such as electric nursing beds and smart home systems, to improve the independence and quality of life of individuals with physical disabilities.

## Figures and Tables

**Table 1:** Names, notations, descriptions, and expressions of ten candidate time-domain features.

Feature Name	Notation	Description	Mathematical Expression
Peak-to-Trough Amplitude	A	Difference between the maximum and minimum of the waveform	A=max(x)−min(x)
Peak-to-Trough Distance	D	Absolute distance (in samples) between the positions of maximum and minimum	D=|argmax(x)−argmin(x)|
Slope of Peak-Trough Segment	S	Slope of the line segment connecting the maximum and minimum	$S=\frac{A}{D}$
Mean Squared Difference of Peak-Trough Segment	E	Mean squared difference of consecutive points within the peak-trough segment	$E=\frac{1}{D-1}\sum_{i=min(argmax(x),argmin(x))}^{max(argmax(x),argmin(x))-1}{\left(x_{i+1}-x_{i}\right)}^{2}$
Skewness	$\gamma_{1}$	Asymmetry of waveform distribution	$\gamma_{1}=\frac{\frac{1}{n}\sum_{i=1}^{n}{\left(x_{i}-\bar{x}\right)}^{3}}{{\left(\frac{1}{n}\sum_{i=1}^{n}{\left(x_{i}-\bar{x}\right)}^{2}\right)}^{\frac{3}{2}}}$
Kurtosis	$\gamma_{2}$	Sharpness of the waveform distribution	$\gamma_{2}=\frac{\frac{1}{n}\sum_{i=1}^{n}{\left(x_{i}-\bar{x}\right)}^{4}}{{\left(\frac{1}{n}\sum_{i=1}^{n}{\left(x_{i}-\bar{x}\right)}^{2}\right)}^{2}}$
Zero-Crossing Rate	Z	Number of times the waveform crosses its mean	$Z=\sum_{i=1}^{n-1}1((x_{i}-\bar{x})(x_{i+1}-\bar{x})<0)$
Energy Ratio of Peak-Trough Segment	R	Ratio of energy in the peak-trough segment to total waveform energy	$R=\frac{\sum_{i=min(argmax(x),argmin(x))}^{max(argmax(x),argmin(x))}{x_{i}}^{2}}{\sum_{j=1}^{n}{x_{j}}^{2}}$
Peak Value	$P_{max}$	Maximum value of the waveform	$P_{max}=max(x)$
Trough Value	$P_{min}$	Minimum value of the waveform	$P_{min}=min(x)$

**Table 3:** Performance metrics for different computer interaction tasks.

Patient Characteristic	Operation Accuracy (%)	Average Operation Time (s)
News reading	88.85±5.03	3.47 ± 0.45
Video playback	87.80 ± 8.23	3.32 ± 0.78
Character spelling	85.32 ± 11.87	3.62 ± 0.82
Overall	87.53 ± 4.92	3.49 ± 0.49

**Table 4:** Correlations between patient characteristics and system performance (* p<0.05).

Patient Characteristic	Operation Accuracy	Average Operation Time	ITR
Fugl-Meyer score	0.448 *	0.220	0.439 *
Age	0.183	0.404	0.080
Disease duration	0.129	0.216	0.105
Brunnstrom upper extremity	0.333	0.219	0.256
Brunnstrom-hand	0.153	0.144	0.177

## Data Availability

The datasets generated and/or analyzed during the current study are not publicly available due to privacy and ethical restrictions, but are available from the corresponding author on reasonable request.

## Abbreviations

The following abbreviations are used in this manuscript.

ALS	amyotrophic lateral sclerosis
BCI	brain–computer interface
DRL	driven right leg
EEG	electroencephalography
EOG	electrooculography
FMA	Fugl-Meyer assessment
FP2	frontal pole 2 (EEG electrode position)
Hz	Hertz
IMU	inertial measurement unit
ITR	information transfer rate
NASA-TLX	National Aeronautics and Space Administration Task Load Index
PC	personal computer
PSD	power spectral density
STFT	short-time Fourier transform
